# The Role of Cardiac Biomarkers in Evaluating Takotsubo Cardiomyopathy: A Systematic Review

**DOI:** 10.7759/cureus.86168

**Published:** 2025-06-16

**Authors:** Pranavi Kilaru, Pranati Kilaru, Shreya Garikipaty

**Affiliations:** 1 Internal Medicine, Mamata Academy of Medical Sciences, Hyderabad, IND; 2 Internal Medicine, Apollo Institute of Medical Sciences and Research, Hyderabad, IND

**Keywords:** apical ballooning syndrome, biomarkers, broken heart syndrome, stress cardiomyopathy, takotsubo cardiomyopathy

## Abstract

Takotsubo cardiomyopathy (TTS) is an acute, reversible cardiac condition marked by transient wall motion abnormalities of the left ventricle, typically triggered by intense emotional or physical stress. Despite being first described in 1990, TTS remains relatively obscure due to limited understanding of its pathophysiology. Clinically, it mimics acute coronary syndrome (ACS), presenting with symptoms such as chest pain, shortness of breath, and ECG changes like ST-segment deviations. This resemblance often leads to initial misdiagnosis and treatment with antiplatelet and anticoagulant therapy - interventions that lack proven efficacy in managing TTS. Given the diagnostic challenges posed by the clinical overlap between TTS and ACS and the absence of disease-specific diagnostic markers, several researchers have investigated the potential role of cardiac biomarkers in distinguishing between the two conditions. This systematic review aims to explore the diagnostic utility of cardiac biomarkers in evaluating TTS. We conducted a systematic literature search using PubMed and Google Scholar, focusing on studies published between 2013 and August 10, 2023. Inclusion criteria were limited to human studies published in English with freely available full texts that evaluated the diagnostic performance of cardiac biomarkers in TTS. A total of eight studies met the criteria and were included for in-depth analysis. These studies examined a range of biomarkers, including established ones such as troponin, B-type natriuretic peptide (BNP), N-terminal proBNP, and creatine kinase-myocardial band (CK-MB), as well as emerging markers like copeptin and microRNAs (miRNAs). Collectively, the studies analyzed data from 3,602 individuals. Troponin showed limited diagnostic accuracy in differentiating TTS from ACS. In contrast, BNP exhibited stronger discriminatory ability, both alone and when used in combination with other markers. The combined use of BNP, troponin, and CK-MB enhanced diagnostic performance. Furthermore, emerging biomarkers such as copeptin and miRNAs demonstrated promising potential due to their early release patterns, association with stress-response mechanisms, and distinct expression profiles, offering new avenues for improving diagnostic accuracy. This review underscores the potential diagnostic value of both established and emerging cardiac biomarkers in identifying TTS. The findings support further research into novel biomarkers, which may ultimately lead to earlier and more accurate diagnosis, reduce inappropriate treatment, and enable more targeted and timely clinical interventions.

## Introduction and background

Takotsubo cardiomyopathy (TTS), also known as broken heart syndrome, apical ballooning syndrome, or stress cardiomyopathy, is an acute and transient cardiac condition characterized by wall motion abnormalities. It was first described in 1990 by Sato et al. [1]. The term “Takotsubo” refers to a Japanese octopus trap, which mirrors the condition’s distinctive echocardiographic appearance - normal contraction at the base of the heart with ballooning at the apex [2]. The syndrome is typically triggered by intense emotional or physical stress, such as bereavement, arguments, trauma, childbirth, or critical illness, hence the rationale behind its various alternative names [2].

The pathogenesis of TTS is believed to involve a surge of catecholamines due to sudden sympathetic nervous system activation, often in response to emotional or physical stress. These episodes are classified as primary TTS (emotionally triggered) or secondary TTS (triggered by physical illness or trauma) [3]. The catecholamine excess leads to transient myocardial stunning, most commonly affecting the left ventricular apex.

Recent studies have also emphasized the role of coronary microvascular dysfunction and neurohormonal dysregulation, including impaired endothelial function and altered adrenergic signaling, as contributory mechanisms that may prolong or worsen myocardial dysfunction in TTS [3]. This pathophysiological combination results in the hallmark apical ballooning and transient reduction in cardiac output observed in affected patients. Fortunately, as sympathetic overactivation resolves, ventricular function generally recovers, and the condition is typically reversible [3].

Epidemiologically, postmenopausal women, particularly those over the age of 55, have nearly a fivefold increased risk of developing TTS [3]. This heightened susceptibility is thought to be linked to declining estrogen levels, which can enhance sympathetic reactivity and impair endothelial function. Clinically, TTS presents with chest pain, dyspnea, palpitations, or syncope - symptoms that closely mimic acute coronary syndrome (ACS), often making initial diagnosis difficult [3]. Because of this overlap and the absence of disease-specific diagnostic tools, the diagnostic utility of cardiac biomarkers in distinguishing TTS from ACS has become an increasingly important area of research.

Diagnosis

Suspected stress cardiomyopathy should be considered in adults, especially postmenopausal women, who present with signs of an ACS, particularly when clinical features and electrocardiographic changes appear disproportionate to the level of cardiac biomarker elevation [4]. A definitive diagnosis of TTS requires the exclusion of other conditions through coronary angiography (CAG) due to the indistinguishable presentation from acute coronary disease [4].

Several diagnostic frameworks have been proposed, most notably the Revised Mayo Clinic Diagnostic Criteria and the International Takotsubo Diagnostic Criteria (InterTAK Diagnostic Criteria) [3]. The Revised Mayo Clinic Diagnostic Criteria place particular emphasis on imaging findings, specifically, transient left ventricular wall motion abnormalities that extend beyond a single coronary vascular territory. These criteria also require the absence of obstructive coronary artery disease, pheochromocytoma, and myocarditis [3]. These are outlined in Table 1 [5].

**Table 1:** Revised Mayo Clinic Diagnostic Criteria Data adapted from Boyd and Solh (2020) [5]

Serial no.	Criterion
1	Transient dyskinesis of the midsegments of the left ventricle
2	Wall motion abnormalities that extend beyond the distribution of a single epicardial vessel
3	Absence of obstructive coronary artery disease or acute plaque rupture
4	New electrocardiographic abnormalities or a modest elevation in troponin levels
5	No evidence of pheochromocytoma or myocarditis

In contrast, the InterTAK Diagnostic Criteria incorporate a broader range of clinical variables, such as emotional or physical stressors, psychiatric or neurologic comorbidities, and specific electrocardiographic findings (e.g., ST-segment changes and QT prolongation) [3]. These elements are used to generate a diagnostic score that supports early risk stratification, particularly in settings where invasive imaging is not immediately available. A summary of the InterTAK scoring system is presented in Table 2 [4].

**Table 2:** International Takotsubo Diagnostic Criteria (InterTAK Diagnostic Criteria) Diagnosis (cutoff value (range 0-100)): a score ≥70 indicates a high probability of TTS; a score <70 suggests a lower probability. TTS: Takotsubo cardiomyopathy Data adapted from Medina de Chazal et al. (2018) [4]

Criteria	Points
Female	25
Emotional trigger	24
Physical trigger	13
Absent ST-segment depression	12
Psychiatric disorders	11
Neurologic disorders	9
QT prolongation	6

Investigations

A diagnostic algorithm for TTS was proposed by the International Takotsubo Registry Group in their 2018 expert consensus statement, published in the European Heart Journal [6]. The initial evaluation of a suspected case begins with an ECG. If ST-segment elevation is observed, the patient should undergo urgent CAG to rule out acute myocardial infarction (AMI) [6]. In cases without ST-segment elevation or with atypical presentations, the InterTAK Diagnostic Score may be applied as an early screening tool. This score combines clinical and ECG parameters to estimate the probability of TTS. A score ≥70 suggests a high likelihood of TTS and supports the use of noninvasive imaging, such as transthoracic echocardiography (TTE) or coronary computed tomography angiography (CCTA), as an initial approach in hemodynamically stable patients [6].

If echocardiography reveals the typical apical ballooning pattern and coronary artery disease is excluded via CCTA or CAG, a diagnosis of TTS can be established. However, in the absence of apical ballooning, further evaluation using invasive CAG may be required. In unstable patients, or when complications are suspected, such as cardiogenic shock, intracardiac thrombus, or ventricular rupture, immediate imaging via TTE or CAG is essential. When coronary artery disease has been excluded and apical ballooning is present, but “red flags” for myocarditis are noted (e.g., fever, recent viral illness, elevated inflammatory markers, eosinophilia, or pericardial effusion), cardiac magnetic resonance imaging (CMR) should be performed to confirm or exclude myocarditis [6]. If CMR findings are normal and follow-up echocardiography shows resolution of wall motion abnormalities, the diagnosis of TTS is further supported [6].

**Figure 1:**
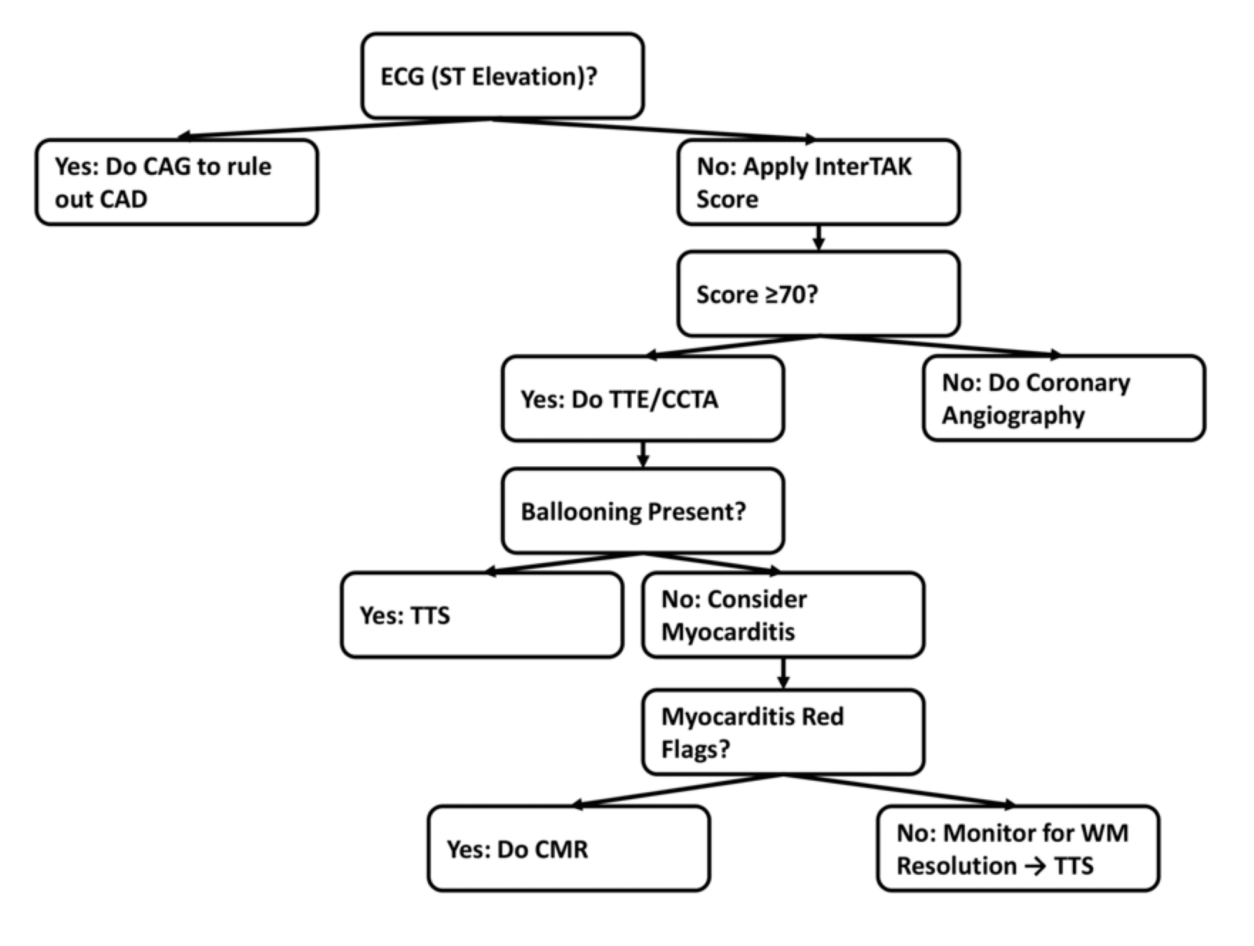
Diagnostic algorithm for TTS (adapted from the 2018 InterTAK consensus statement) CAD: coronary artery disease; CAG: coronary angiography; CCTA: coronary computed tomography angiography; CMR: cardiac magnetic resonance imaging; TTE: transthoracic echocardiography; TTS: Takotsubo cardiomyopathy; WM: wall motion Adapted from Ghadri et al. (2018) [6]

A summarized diagnostic workflow, adapted from the 2018 International Takotsubo Registry consensus, is presented in Figure 1 for clarity.

Cardiac biomarkers play a critical role in the diagnosis and differentiation of TTS, particularly in distinguishing it from ACS. These biomarkers are endogenous substances released into the bloodstream in response to myocardial injury, stress, or necrosis, and are essential for the early detection and clinical management of various cardiovascular disorders [7].

Key biomarkers include indicators of myocardial necrosis - such as myoglobin, creatine kinase-myocardial band (CK-MB), and cardiac troponins I and T - as well as markers of hemodynamic stress, namely B-type natriuretic peptide (BNP) and N-terminal proBNP (NT-proBNP). BNP and NT-proBNP are both cleaved from the same precursor molecule, proBNP. While NT-proBNP has a longer half-life and is excreted renally, making it more sensitive to renal dysfunction, BNP is relatively unaffected by renal status.

In recent years, novel biomarkers have garnered growing interest. MicroRNAs (miRNAs) - small, noncoding RNAs that regulate gene expression - have shown promise due to their tissue specificity and remarkable stability in circulation. These characteristics make them attractive candidates for diagnostic use in TTS and other cardiac conditions [7]. Likewise, copeptin, a stable peptide fragment derived from the precursor of vasopressin, has gained recognition for its early response to physiological stress and may improve diagnostic sensitivity when used alongside established biomarkers [7,8].

In the context of TTS, cardiac biomarkers exhibit distinct response profiles that underscore their diagnostic relevance. Cardiac troponin is elevated in over 90% of TTS cases; however, both troponin and creatine kinase (CK) levels are often disproportionately low relative to the degree of regional wall motion abnormalities and global cardiac dysfunction [6]. While initial troponin concentrations may resemble those in ACS, peak values are typically lower in TTS. Nonetheless, elevated admission troponin levels are associated with worse in-hospital outcomes [6], although CK tends to show only modest increases.

Serum natriuretic peptides such as BNP and NT-proBNP are almost universally elevated in TTS and correlate more consistently with left ventricular dysfunction. These markers typically peak within 24-48 hours after symptom onset and gradually normalize over several weeks to months [6]. NT-proBNP levels, in particular, are influenced by sympathetic activation, systemic inflammation (e.g., CRP), and the severity of systolic dysfunction. Studies suggest that BNP and NT-proBNP offer superior diagnostic value compared to troponin in differentiating TTS from ACS, and current guidelines recommend their measurement in all suspected TTS cases. Notably, elevated natriuretic peptide levels have been integrated into updated diagnostic criteria, and low NT-proBNP levels at admission may indicate a favorable prognosis [6].

Among the emerging biomarkers, copeptin has shown particular promise. Its early and robust rise in response to hemodynamic stress makes it a useful early marker, especially when used in combination with NT-proBNP. In fact, the copeptin/NT-proBNP ratio has demonstrated strong discriminatory power in differentiating TTS from AMI [9].

Similarly, miRNAs have shown diagnostic potential in cardiovascular disease due to their specific expression patterns and stability in blood. A study by Jaguszewski et al. identified a TTS-specific miRNA signature, comprising miR-16, miR-26a, miR-1, and miR-133a, capable of differentiating TTS from ACS. Although further studies are needed to validate their prognostic utility, these miRNAs hold substantial promise for enhancing diagnostic accuracy [10].

This review aims to examine the evolving role of cardiac biomarkers in the evaluation of TTS, highlighting the diagnostic performance of both established and emerging biomarkers. By analyzing findings from clinical trials, systematic reviews, and observational studies, we seek to enhance understanding and improve the diagnostic approach to this unique and often misdiagnosed cardiac condition.

## Review

Methods

This review focuses on clinical studies exploring the role of cardiac biomarkers in TTS. It adheres to the 2020 Preferred Reporting Items for Systematic reviews and Meta-Analyses (PRISMA) guidelines [11] and includes only data extracted from previously published studies, eliminating the need for ethical approval.

**Figure 2:**
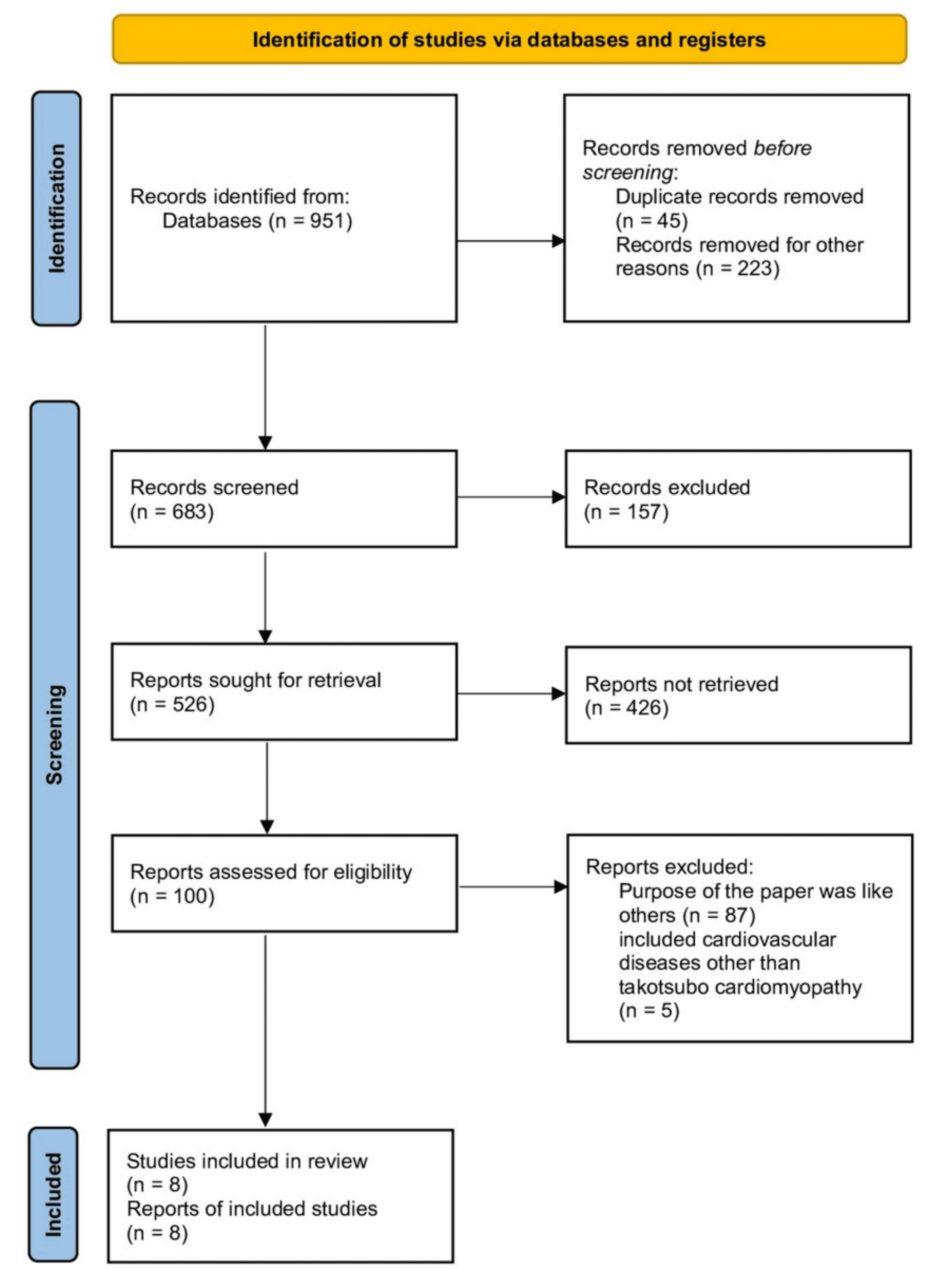
PRISMA flow diagram illustrating the search strategy and study selection process for the systematic review PRISMA: Preferred Reporting Items for Systematic reviews and Meta-Analyses

Figure 2 presents the PRISMA flow diagram, which outlines the study screening and selection process.

Although this review meets the criteria for a systematic review, a meta-analysis was not conducted due to substantial heterogeneity across the included studies. Differences in study design, biomarker assay methods, timing of sample collection, and outcome reporting prevented meaningful statistical pooling. As a result, findings were synthesized using a narrative approach.

This method ensures scientific rigor and transparency, and future updates may include meta-analytic techniques should more standardized data become available.

Systematic Literature Search and Study Selection

A comprehensive literature search was performed using PubMed (including Medline) and Google Scholar to identify studies investigating the role of cardiac biomarkers in TTS. Additionally, the reference lists of relevant review articles, editorials, and commentaries were screened to capture any potentially overlooked studies.

The screening process was carried out by three independent reviewers (PK, PK, and SG). Titles and abstracts were initially reviewed independently by two reviewers, and any discrepancies were resolved through discussion and consensus among all three. Blinding was not employed during the screening or data extraction phases, and inter-rater agreement was not statistically evaluated using measures such as the kappa coefficient.

In Google Scholar, only the first 200 results per search term were reviewed, sorted by date and relevance. Gray literature was excluded, as the review focused solely on peer-reviewed clinical studies to ensure scientific validity and reproducibility.

Inclusion and Exclusion Criteria

The inclusion and exclusion criteria were developed to select high-quality, relevant studies, as summarized in Table 3. Eligible study designs included randomized controlled trials (RCTs), cohort studies, case-control studies, and cross-sectional studies that evaluated cardiac biomarkers in the context of TTS.

**Table 3:** Criteria adopted during the literature search process

Inclusion criteria	Exclusion criteria
Human studies	Animal studies
Publications from 2013 to 2023	Non-English language articles
Peer-reviewed, full-text articles available	Studies not accessible through institutional access
Study types: RCTs, cohort, case-control, cross-sectional studies, systematic reviews, and meta-analyses	Letters to the editor

Articles requiring purchase were excluded to maintain accessibility and transparency in data sourcing. While this may introduce selection bias, the decision reflects practical constraints related to access and reproducibility. We acknowledge this as a limitation of the review and recommend broader access in future systematic reviews or meta-analyses to mitigate this bias.

Search Strategy

The search was conducted using PubMed (including Medline) and Google Scholar. Filters applied included a publication date range from 2013 to 2023, English language, and human studies, in alignment with the predefined inclusion criteria. Only peer-reviewed, full-text articles were considered eligible for inclusion.

Keywords used in the search included cardiac biomarkers, Takotsubo cardiomyopathy, stress cardiomyopathy, apical ballooning syndrome, and broken heart syndrome. Additionally, the Medical Subject Headings (MeSH) approach was applied to create a comprehensive search strategy in PubMed (including Medline), incorporating MeSH terms such as apical ballooning syndrome, broken heart syndrome, and stress cardiomyopathy.

Table 4 provides a detailed summary of the search strategy employed.

**Table 4:** Search strategy, search engines used, and number of results retrieved

Database	Search strategy	Search results
PubMed (including MEDLINE)	“Takotsubo cardiomyopathy” AND (“cardiac biomarkers” OR “apical ballooning syndrome” OR “broken heart syndrome” OR “stress cardiomyopathy”)	151
Google Scholar	“Takotsubo cardiomyopathy” AND “cardiac biomarkers”	18,500
	“Stress cardiomyopathy” AND “cardiac biomarkers”	16,900
	“Apical ballooning syndrome” AND “cardiac biomarkers”	12,700
	“Broken heart syndrome” AND “cardiac biomarkers”	16,500

Quality Appraisal

To ensure the credibility and reliability of the included studies, various standardized tools were used to assess methodological quality. For systematic reviews and meta-analyses, the PRISMA checklist was applied. Randomized clinical trials were evaluated using the Cochrane Risk of Bias Tool, while nonrandomized clinical trials were assessed using the Newcastle-Ottawa Scale.

Additionally, the Critical Appraisal Skills Programme checklist was used for general quality evaluation. To avoid misclassification, the Scale for the Assessment of Narrative Review Articles (SANRA) was applied to appraise narrative reviews. A summary of the quality assessment results is presented in Table 5.

**Table 5:** Quality appraisal tools used PRISMA: Preferred Reporting Items for Systematic reviews and Meta-Analyses; RCT: randomized controlled trial; SANRA: Scale for the Assessment of Narrative Review Articles

Quality appraisal tool	Type of study
Cochrane risk of bias tool	RCT
Newcastle-Ottawa Scale	Non-RCTs and observational studies
PRISMA checklist	Systematic reviews
SANRA checklist	Narrative reviews or studies without a defined methodology

Results

After searching two selected databases - PubMed (including Medline) and Google Scholar - we initially identified a total of 64,751 articles. To maintain feasibility while ensuring comprehensive coverage, Google Scholar screening was limited to the first 20 pages (10 articles per page) per search query, yielding a total of 800 results. This approach aligns with previous systematic reviews that found later pages often include duplicates or less relevant studies. All results were sorted by relevance and filtered by date (2013-2023), consistent with our predefined inclusion criteria.

The PubMed search returned 151 results, bringing the total number of articles identified before screening to 951. Each article was carefully reviewed using specific inclusion and exclusion criteria. We excluded 268 articles due to duplication or irrelevant titles and abstracts. Of the remaining 683, an additional 157 were excluded based on full-text review. From the resulting 526 articles, 518 were excluded as they did not meet the criteria for this review.

A final set of eight articles passed our quality appraisal and met all inclusion standards. These eight studies were included in the systematic review. A detailed description of each study is presented in Table 6.

**Table 6:** Summary of the findings from the selected studies ACS: acute coronary syndrome; AMI: acute myocardial infarction; BNP: B-type natriuretic peptide; CK: creatine kinase; CK-MB: creatine kinase-myocardial band; EF: ejection fraction; hs-TnT: high-sensitivity cardiac troponin T; MI: myocardial infarction; miRNA: microRNA; NSTEMI: non-ST elevated myocardial infarction; NT-proBNP: N-terminal pro-B-type natriuretic peptide; STEMI: ST elevated myocardial infarction; TnI: troponin I; TnT: troponin T; TTS: Takotsubo cardiomyopathy

Author and year	Country	Study design	Database used	Conclusion
Budnik et al. (2020) [9]	Europe	Prospective clinical study	PubMed	The serum copeptin/NT-proBNP ratio may assist in noninvasive differentiation between TTS and STEMI; further studies are needed.
Jaguszewski et al. (2014) [10]	Europe	Prospective clinical study	PubMed	A panel of four circulating miRNAs may serve as a molecular signature for distinguishing TTS from STEMI patients.
Templin et al. (2015) [12]	Europe	Retrospective clinical study	PubMed	Certain biomarkers, such as troponin max, CK, and BNP, show significant differences between ACS and TTS.
Doyen et al. (2014) [13]	France	Multicenter prospective clinical study	PubMed	BNP/TnI and BNP levels were helpful in differentiating TTS from STEMI and NSTEMI.
Dagrenat et al. (2020) [14]	France	Prospective clinical study	PubMed	A distinct cardiac biomarker profile exists for TTS, though considerable overlap with ACS remains.
Pirlet et al. (2017) [15]	Europe	Prospective and retrospective clinical study	PubMed	The hs-TnT/CK-MB ratio is a novel parameter that may aid in differentiating TTS from MI.
Budnik et al. (2016) [16]	Poland	Retrospective clinical study	PubMed	NT-proBNP/TnI, NT-proBNP/CK-MB mass, and NT-proBNP/EF ratios can distinguish TTS from STEMI early, with NT-proBNP/TnI being the most accurate.
Randhawa et al. (2014) [17]	USA	Retrospective clinical study	PubMed	Early BNP/TnT and BNP/CK-MB ratios differentiate TTS from AMI more effectively than BNP alone.

Discussion

This systematic review identified eight key clinical studies evaluating the role of cardiac biomarkers in distinguishing TTS from ACS. A central challenge highlighted across these studies is the absence of a single biomarker that can consistently and independently differentiate TTS from ACS. Instead, diagnostic accuracy improves when using biomarker ratios, combinations, or emerging molecular tools.

Troponin I (Trop I)

A 2014 prospective study involving 63 TTS and 90 ACS patients described a characteristic Trop I pattern in TTS: a rapid peak followed by a decline and then a plateau. Trop I was positive on admission in 98.4% of patients, but the peak typically occurred before hospital admission, often resulting in a decreasing trend upon presentation [13]. Similarly, a 2015 retrospective study of 1,750 TTS patients found that while maximum troponin levels were significantly different between TTS and ACS groups, admission values were comparable [12]. These findings confirm that although troponin is often elevated in TTS, it lacks diagnostic specificity when used alone.

BNP

Several studies consistently found higher BNP levels in TTS patients compared to those with ACS. The 2014 prospective study reported significantly elevated BNP in TTS patients versus those with ST-elevated myocardial infarction (STEMI) or non-ST-elevated myocardial infarction (NSTEMI), emphasizing that BNP is often the only natriuretic peptide available in emergency settings [13]. The 2015 retrospective study also found both admission and peak BNP levels to be significantly higher in TTS compared to ACS [12]. Randhawa et al. further supported this trend [17], indicating BNP as a potentially useful individual or combined diagnostic marker.

BNP/Trop I Ratio

The BNP/Trop I ratio emerged as a particularly promising diagnostic tool. In a retrospective study of 1,100 patients, this ratio was calculated at both admission and peak troponin levels. A cutoff value of 39 was reported to identify TTS with 88% sensitivity and 94% specificity. For NSTEMI, a cutoff of 76.5 yielded 66% sensitivity and 79% specificity. Diagnostic accuracy improved when the ratio was calculated at peak values [14]. Another prospective study reported a BNP/Trop I ratio >159 identified TTS with 95.2% sensitivity and 97.9% specificity, while a cutoff of 310 in NSTEMI showed 82.3% sensitivity and 71.4% specificity [13]. These findings support the BNP/Trop I ratio as superior to either marker alone.

CK

The 2015 study noted that CK was not substantially elevated in most TTS cases [12]. Similarly, a prospective study reported lower CK and CK-MB levels in TTS compared to STEMI patients, though levels were similar to those seen in NSTEMI [13]. These findings suggest CK and CK-MB alone are not reliable for distinguishing TTS from ACS.

High-Sensitivity Cardiac Troponin T (hs-TnT)/CK-MB Ratio

A 2017 single-center, dual-phase study investigated the diagnostic utility of the hs-TnT/CK-MB ratio in differentiating TTS from ACS [15]. While hs-TnT, a preferred biomarker for diagnosing AMI, allows earlier detection of low troponin levels [18], it did not differ significantly between TTS and MI in this study. However, CK-MB levels did differ, resulting in a significantly higher hs-TnT/CK-MB ratio in TTS patients across both phases of the study. Cutoff thresholds of 0.015 and 0.017 yielded high sensitivity and specificity, suggesting that this ratio, particularly when combined with other biomarkers, may enhance early diagnostic accuracy.

NT-proBNP and Biomarker Ratios

Both BNP and NT-proBNP are derived from the cleavage of the precursor molecule proBNP, and both serve important roles in the diagnosis of cardiac conditions. Notably, NT-proBNP has a longer half-life than BNP and is primarily cleared through the kidneys, resulting in significantly higher serum levels [19].

CK-MB mass, also known as total CK-MB, refers to the measurement of the total amount of CK-MB in the blood, regardless of its enzymatic activity or subunit composition. A 2021 study demonstrated that measuring CK-MB mass is superior to measuring CK-MB activity in diagnosing AMI [20].

A retrospective study by Budnik et al. (sample size not specified) evaluated NT-proBNP, TnI, CK, and CK-MB mass levels within 12 hours of admission. NT-proBNP levels were significantly higher in patients with TTS, while TnI and CK-MB mass levels were lower. No significant differences were observed in CK levels. Importantly, the NT-proBNP/TnI and NT-proBNP/CK-MB mass ratios were markedly higher in patients with TTS than in those with STEMI. Specifically, the NT-proBNP/TnI ratio was 2235.2 in TTS patients compared to 81.6 in those with ACS, while the NT-proBNP/CK-MB mass ratio was 678.2 in TTS patients versus 27.5 in STEMI patients (p < 0.001), indicating statistical significance. These findings suggest that these ratios can distinguish TTS from STEMI more accurately than NT-proBNP levels alone. The study also reported a higher NT-proBNP/ejection fraction ratio in the TTS group compared to the STEMI group [16].

A separate 2013 study supports these findings, showing that BNP/troponin T and BNP/CK-MB ratios outperform BNP alone in differentiating TTS from ACS [17]. Among the ratios studied, the NT-proBNP/TnI ratio demonstrated the highest diagnostic accuracy [16]. However, BNP may be preferred over NT-proBNP in some cases, as it is less influenced by renal function [13].

Copeptin

Another study by Budnik et al. (2020) investigated a newer cardiac biomarker - copeptin. During myocardial infarction, the stress hormone arginine vasopressin (AVP), also known as antidiuretic hormone, is released in response to stress and hemodynamic changes. Copeptin, a related molecule derived from the C-terminal portion of pre-pro-AVP, has gained attention as a cardiac biomarker due to its greater stability compared to AVP. It is secreted in equimolar amounts with AVP and shows a rapid increase during conditions such as AMI. Copeptin is especially useful when combined with other biomarkers like TnI, as it improves diagnostic accuracy. Additionally, it serves as an indicator of adverse outcomes in patients with ACS. In this context, copeptin effectively acts as a surrogate marker for AVP [21].

In a prospective study conducted in 2020, involving 19 women with TTS and 10 with STEMI, serum copeptin levels were significantly lower in TTS compared to STEMI. The cutoff value for serum copeptin to identify STEMI patients was >1.306 mg/mL, yielding a sensitivity of 90% and a specificity of 79% [9]. The authors referenced a 2012 study that reported that (i) serum copeptin levels were elevated in 50% of patients with TTS, and (ii) patients exhibiting the typical apical ballooning pattern had lower copeptin levels compared to those with an atypical (midventricular) wall motion abnormality. The study also highlighted that although elevated plasma catecholamines are commonly observed in TTS, the rise in stress hormones such as epinephrine, norepinephrine, cortisol, AVP, and copeptin is not specific to cardiac events and can also occur in noncardiac conditions. Moreover, the levels of these stress markers are influenced by the time elapsed since the triggering event. Therefore, measuring stress hormone levels may offer limited diagnostic value in differentiating between ACS and TTS and may not provide substantial insights into the underlying mechanisms of TTS [22].

The study presents conflicting findings regarding copeptin elevation in typical versus atypical TTS wall motion abnormalities, thereby limiting its standalone diagnostic reliability. However, the serum copeptin to NT-proBNP ratio appears to be a promising noninvasive tool for differentiating between TTS and STEMI [9].

miRNAs

miRNAs are noncoding RNA molecules approximately 22 nucleotides long that regulate gene expression by promoting mRNA degradation or inhibiting its translation. A single miRNA can target multiple mRNAs, and each mRNA can, in turn, be regulated by multiple miRNAs, resulting in complex and layered regulatory effects. The human genome encodes about 1,000 miRNAs, with 721 identified so far. Many miRNAs are tissue-specific and play essential roles in both physiological and pathological processes, including cardiovascular disease. Their expression levels are known to change in response to stress.

Circulating miRNAs have been detected in plasma and other bodily fluids, where they are protected from degradation by encapsulation within microvesicles and association with protein complexes. This stability makes them attractive candidates as biomarkers for cardiovascular diseases, as their altered levels have been observed in conditions such as stable coronary artery disease, diabetes, AMI, and heart failure [23,24].

In a study by Jaguszewski et al., the researchers aimed to identify specific circulating miRNAs that could aid in the diagnosis of acute TTS and help distinguish it from AMI. Eight miRNAs were initially selected through profiling and subsequently validated using real-time qRT-PCR in patients with TTS, STEMI, and healthy controls.

The results showed that let-7f, miR-22, and miR-519d were significantly upregulated in patients with TTS. MiR-16 and miR-26a, which are associated with stress and depression, were also found to be dysregulated. MiR-125a-5p, possibly linked to vascular endothelial cells, was downregulated. These miRNAs were evaluated further alongside miR-1 and miR-133a, both of which are well-established markers elevated after MI.

Subsequent analysis revealed that miR-16 and miR-26a were significantly elevated in TTS patients compared to both healthy controls and STEMI patients. Let-7f was also markedly upregulated in TTS patients relative to those with STEMI. Meanwhile, miR-1 and miR-133a were upregulated in both TTS and STEMI patients compared to healthy individuals. A combination of miR-16, miR-26a, miR-1, and miR-133a enhanced diagnostic accuracy by offering a distinct molecular signature, although specific sensitivity and specificity values were not provided [10].

miRNAs have thus emerged as promising, minimally invasive biomarkers that remain stable in circulation and offer the potential for personalized diagnostic profiling based on specific molecular expression patterns [10].

Taken together, copeptin and miRNAs represent two emerging and complementary tools in the diagnostic landscape of TTS. Copeptin provides early-phase biochemical information, while miRNAs reflect gene expression changes in response to stress. Their combined use may enhance diagnostic precision by capturing both systemic and cellular responses.

Synthesis and Limitations

This systematic review highlights that while individual biomarkers such as troponin and CK lack sufficient specificity, certain combinations, particularly the BNP/TnI and NT-proBNP/TnI ratios, consistently demonstrate superior diagnostic accuracy in distinguishing TTS from ACS. Copeptin and its derived ratios, especially when combined with NT-proBNP, also show promise; however, their utility is limited by variability associated with noncardiac stress. Similarly, miRNA signatures represent a novel and noninvasive diagnostic approach, though their application remains in the early stages of investigation.

Several factors complicate the interpretation of these biomarkers. NT-proBNP levels are significantly influenced by renal function, while copeptin levels fluctuate in response to general stress, reducing their cardiac specificity. The diagnostic performance of many markers is also affected by the timing of sample collection, particularly when early troponin peaks are missed, as well as inconsistencies in assay methods, marker availability, and threshold values. Additionally, heterogeneity in study design and small sample sizes limit the comparability and generalizability of findings across studies.

Overall, these findings suggest that future diagnostic protocols may benefit from integrating multimarker ratios or incorporating emerging biomarkers such as miRNAs or copeptin to improve accuracy in differentiating TTS from ACS.

## Conclusions

This systematic review underscores the ongoing need for reliable cardiac biomarkers capable of accurately distinguishing TTS from ACS. Traditional markers such as TnI and CK lack the specificity required for this differentiation. However, several biomarker ratios, particularly BNP/TnI, NT-proBNP/TnI, and NT-proBNP/CK-MB mass, have demonstrated improved diagnostic accuracy across multiple studies. Among these, the NT-proBNP/TnI ratio consistently exhibited the highest sensitivity and specificity, positioning it as one of the most promising candidates for near-term clinical implementation. Copeptin, while potentially valuable for early-phase discrimination between TTS and STEMI, has shown inconsistent diagnostic performance across studies. Its levels can be elevated in response to noncardiac stress and are influenced by the timing of sample collection, limiting its reliability as a standalone marker. As such, further prospective validation is required before it can be routinely incorporated into clinical practice.

miRNAs such as miR-16 and miR-26a, which demonstrate distinct expression profiles in TTS compared to ACS, represent a novel class of biomarkers with considerable diagnostic potential. Their stability in circulation and stress-specific expression patterns offer promising opportunities for future research and potential clinical translation. Importantly, most studies included in this review were limited by small sample sizes, lack of methodological standardization, and insufficient long-term validation. These limitations highlight the need for larger, multicenter studies. Continued exploration of multimarker strategies, particularly those that combine well-established and emerging biomarkers, may significantly improve diagnostic accuracy, reduce the risk of misdiagnosis, and support more informed therapeutic decision-making in patients with TTS.

## References

[1] Sato H, Tateishi H, Uchida T (1990). Takotsubo-type cardiomyopathy due to multivessel spasm. Clinical Aspect of Myocardial Injury: From Ischemia to Heart Failure.

[2] SVH Heart Health. (n.d Takotsubo cardiomyopathy. https://www.svhhearthealth.com.au/conditions/takotsubo-cardiomyopathy.

[3] Ghadri JR, Wittstein IS, Prasad A (2018). International expert consensus document on Takotsubo syndrome (part I): clinical characteristics, diagnostic criteria, and pathophysiology. Eur Heart J.

[4] Medina de Chazal H, Del Buono MG, Keyser-Marcus L, Ma L, Moeller FG, Berrocal D, Abbate A (2018). Stress cardiomyopathy diagnosis and treatment: JACC state-of-the-art review. J Am Coll Cardiol.

[5] Boyd B, Solh T (2020). Takotsubo cardiomyopathy: review of broken heart syndrome. JAAPA.

[6] Ghadri JR, Wittstein IS, Prasad A (2018). International expert consensus document on Takotsubo syndrome (part II): diagnostic workup, outcome, and management. Eur Heart J.

[7] Dasgupta A & Wahed A Clinical chemistry, immunology and laboratory quality control. https://www.sciencedirect.com/topics/medicine-and-dentistry/cardiac-marker.

[8] Jacob R, Khan M (2018). Cardiac biomarkers: what is and what can be. Indian J Cardiovasc Dis Women WINCARS.

[9] Budnik M, Białek S, Peller M (2020). Serum copeptin and copeptin/NT-proBNP ratio — new tools to differentiate Takotsubo syndrome from acute myocardial infarction. Folia Med Cracov.

[10] Jaguszewski M, Osipova J, Ghadri JR (2014). A signature of circulating microRNAs differentiates Takotsubo cardiomyopathy from acute myocardial infarction. Eur Heart J.

[11] Liberati A, Altman DG, Tetzlaff J (2009). The PRISMA statement for reporting systematic reviews and meta-analyses of studies that evaluate health care interventions: explanation and elaboration. PLoS Med.

[12] Templin C, Ghadri JR, Diekmann J (2015). Clinical features and outcomes of Takotsubo (stress) cardiomyopathy. N Engl J Med.

[13] Doyen D, Moceri P, Chiche O (2014). Cardiac biomarkers in Takotsubo cardiomyopathy. Int J Cardiol.

[14] Dagrenat C, Von Hunolstein JJ, Matsushita K (2020). Value of cardiac biomarkers in the early diagnosis of Takotsubo syndrome. J Clin Med.

[15] Pirlet C, Pierard L, Legrand V, Gach O (2017). Ratio of high-sensitivity troponin to creatine kinase-MB in Takotsubo syndrome. Int J Cardiol.

[16] Budnik M, Kochanowski J, Piatkowski R (2016). Simple markers can distinguish Takotsubo cardiomyopathy from ST segment elevation myocardial infarction. Int J Cardiol.

[17] Randhawa MS, Dhillon AS, Taylor HC, Sun Z, Desai MY (2014). Diagnostic utility of cardiac biomarkers in discriminating Takotsubo cardiomyopathy from acute myocardial infarction. J Card Fail.

[18] Xu RY, Zhu XF, Yang Y, Ye P (2013). High-sensitive cardiac troponin T. J Geriatr Cardiol.

[19] Weber M, Hamm C (2006). Role of B-type natriuretic peptide (BNP) and NT-proBNP in clinical routine. Heart.

[20] Kumar NS, Rajendran S, Nanda SK, Christopher M, Kandasamy R (2021). A comparative study on the diagnostic utility of creatine kinase-MB (Myocardial Band) mass estimation over its activity measurement in patients with acute myocardial infarction in a tertiary care hospital in Puducherry. J Evid Based Med Healthc.

[21] Mu D, Cheng J, Qiu L, Cheng X (2022). Copeptin as a diagnostic and prognostic biomarker in cardiovascular diseases. Front Cardiovasc Med.

[22] Burgdorf C, Schubert A, Schunkert H, Kurowski V, Radke PW (2012). Release patterns of copeptin and troponin in Tako-Tsubo cardiomyopathy. Peptides.

[23] Widera C, Gupta SK, Lorenzen JM (2011). Diagnostic and prognostic impact of six circulating microRNAs in acute coronary syndrome. J Mol Cell Cardiol.

[24] D'Alessandra Y, Devanna P, Limana F (2010). Circulating microRNAs are new and sensitive biomarkers of myocardial infarction. Eur Heart J.

